# Data-Driven Decision Making and Proactive Citizen–Scientist Communication: A Cross-Sectional Study on COVID-19 Vaccination Adherence

**DOI:** 10.3390/vaccines9121384

**Published:** 2021-11-24

**Authors:** Emil Syundyukov, Martins Mednis, Linda Zaharenko, Eva Pildegovica, Ieva Danovska, Svjatoslavs Kistkins, Abraham Seidmann, Arriel Benis, Valdis Pirags, Lilian Tzivian

**Affiliations:** 1 Longenesis Ltd., Zaubes str. 9A-23, LV-1013 Riga, Latvia; m-mednis@longenesis.com (M.M.); lz@longenesis.com (L.Z.); eva@skrinings.lv (E.P.); id@longenesis.com (I.D.); 2 Faculty of Computing, University of Latvia, Raina Boulevard 19, LV-1050 Riga, Latvia; 3 School of Health in Social Science, Old Medical School, University of Edinburgh, Edinburgh EH8 9AG, UK; 4 Department of Internal Medicine, Paul Stradins Clinical University Hospital, Pilsonu str. 13, LV-1002 Riga, Latvia; svjatoslavs.kistkins@stradini.lv (S.K.); valdis.pirags@stradini.lv (V.P.); 5 Questrom Business School, Boston University, Boston, MA 02215, USA; avis@bu.edu; 6 Health Analytics and Digital Health, Digital Business Institute, Boston University, Boston, MA 02215, USA; 7 Faculty of Industrial Engineering and Technology Management, Holon Institute of Technology, Holon 5810201, Israel; arrielb@hit.ac.il; 8 Faculty of Digital Technologies in Medicine, Holon Institute of Technology, Holon 5810201, Israel; 9 Faculty of Medicine, University of Latvia, Jelgavas str. 3, LV-1004 Riga, Latvia; liliana.civjane@lu.lv

**Keywords:** digital health, data-driven communication, COVID-19 vaccination readiness, coronavirus, SARS-CoV-2, immunization programs, vaccination refusal, vaccination hesitancy, health communication, web-based survey

## Abstract

Due to the severe impact of COVID-19 on public health, rollout of the vaccines must be large-scale. Current solutions are not intended to promote an active collaboration between communities and public health researchers. We aimed to develop a digital platform for communication between scientists and the general population, and to use it for an exploratory study on factors associated with vaccination readiness. The digital platform was developed in Latvia and was equipped with dynamic consent management. During a period of six weeks 467 participants were enrolled in the population-based cross-sectional exploratory study using this platform. We assessed demographics, COVID-19-related behavioral and personal factors, and reasons for vaccination. Logistic regression models adjusted for the level of education, anxiety, factors affecting the motivation to vaccinate, and risk of infection/severe disease were built to investigate their association with vaccination readiness. In the fully adjusted multiple logistic regression model, factors associated with vaccination readiness were anxiety (odds ratio, OR = 3.09 [95% confidence interval 1.88; 5.09]), feelings of social responsibility (OR = 1.61 [1.16; 2.22]), and trust in pharmaceutical companies (OR = 1.53 [1.03; 2.27]). The assessment of a large number of participants in a six-week period show the potential of a digital platform to create a data-driven dialogue on vaccination readiness.

## 1. Introduction

The World Economic Forum’s Global Risks Report 2021 has specified infectious diseases as the main threat for this year. The report indicates that COVID-19 has led to widespread loss of life and increased wealth inequality, and efforts to combat it have taken resources away from the research and treatment of other serious public health issues [1]. Scientists and clinicians are intensively investigating the epidemiology and pathology of the coronavirus, and preparing new vaccines and treatments to return life to the pre-COVID situation [2].

The World Health Organization (WHO) announced COVID-19 as a global pandemic on 11 March 2020. Since then, governments and public institutions worldwide have been faced with tough decisions concerning health security policies in their respective countries. The quick and efficient engagement of the general public and of respondents in the implementation of official guidelines for the prevention of SARS-CoV-2 transmission has been identified as one of the key factors for successfully dealing with pandemics [3]. From the beginning of the pandemic to the end of May 2021, the total number of cases in Latvia has reached 133,098, and 125,712 have recovered during this period. The first two cases were identified on 8 March 2020 [4]. Most of the cases (*n* = 46,040) were discovered in Riga, the capital of Latvia, which is also the place with the highest population density [5]. The state performs most of the tests in social care institutions and shelters, so most COVID-19 cases were found there. In addition, on 12 March 2020, a state of emergency was declared in Latvia [6]. According to official statistics, the total number of deaths from COVID-19 was 2370 as of 31 May 2021 [4]. According to the Latvian Centre of Disease Prevention and Control [7], the percentage of the population that had been vaccinated was 21.2% as of the end of May 2021.

The number of COVID-19 tests performed daily in Latvia increased in September 2021 with the beginning of the school year, since school children are now being tested weekly. Before 1 September 2021, the cumulative number of tests was 134,289, while on 8 November 2021, the cumulative rate of COVID-19 tests in Latvia was 2.62 million per million people in the population. This testing rate is comparable with the number of tests in other Baltic countries (Lithuania 2.18 million, Estonia 1.64 million per million). It is also comparable with some other European countries (e.g., the testing rate in Finland was 1.35 million per million.) [8]. Nevertheless, in the latest period, Latvia had a high incidence of COVID-19 which exceeded the mean EU number of cases per million of the population. For example, on 8 November 2021, there were 943.95 new cases per million in Latvia vs. 321.16 new cases per million on average in the EU. Latvia at this time also had a much higher mortality rate than in the EU. For example, by 8 November 2021, Latvia had 18.82 daily deaths per million, while in the EU this rate was about 4.75 per million [8]. Although the COVID-19 infection and mortality rates do change over time, the leadership in Latvia has a high demand for a fast and effective vaccination process covering a significant percentage of participants.

In Latvia, the national health expenditures are about the lowest in the European Union (EU), amounting to only 5.8% of the GDP (in comparison with the mean EU expenditures of 8.3% in 2019). Physician density in Latvia is 3.19/1000 people, and hospital bed density is 3.2 beds/1000 people in comparison with the EU mean of 3.60/1000 people and 5.38/1000, respectively (data for 2018) [9]. Thus, Latvia is in a relatively weak position with regard to its medical supply and readiness to face challenges like COVID-19. Therefore, there is a high demand for a fast and effective vaccination process with a high percentage of participants. In this case, if technological solutions can help health authorities to recognize specific groups of people that hesitate regarding vaccination or identify factors related to a reduced readiness for vaccination, authorities can concentrate their efforts in predefined directions. Such instruments are now being used to capture the public health response to the COVID-19 pandemic in a safe and effective way, expanding from epidemiological surveillance, case identification, interruption of community transmission, and public communication to day-to-day medical care. An excellent example is the NHS COVID-19 App, which tracks proximity between app users and informs their recent contacts when users test positive for COVID-19. A study estimates that use of this app by 16.5 million users (28% of UK population) has prevented between 248,000 and 594,000 cases [10]. A similar governmental app, Stop COVID, was implemented in Latvia. It now has more than 300,000 downloads, which makes the user base approximately 17% of the whole targeted national population (age 18+) [11].

From the moment when COVID-19 vaccines were discovered and available for the general public, vaccine hesitance emerged as the main social barrier to mass vaccinations. Vaccine hesitance is not a new problem. An explanation of activities that should be planned to tackle it were already proposed before the pandemic [12,13]. Several studies have concluded that about half of the eligible public are willing to get the COVID-19 inoculation [14]; however, about a third of them are still undecided and need more convincing [15]. The major potential reasons that can cause vaccine uncertainty in more than 80% of unvaccinated people can be concerns regarding potential side effects, safety, and an overall lack of pertinent information [16]. For example, the lack of knowledge on side effects and vaccine manufacturing conditions was mentioned as the main factor for refusing to vaccinate 1062 college students in North Carolina, USA [17]. The most essential problem mentioned in a recent OECD report on COVID-19 vaccination was a lack of public trust in vaccines and their efficacy [18]. This problem can be solved by communication transparency with the public; being forthcoming with the publication of research on vaccine efficacy and providing clear governmental communications about anticipated vaccination strategies [19]. Other factors that can play a role in the decision of individuals to vaccinate are religious and cultural issues; however, according to some studies, these factors are not the most prominent ones and should be considered mostly in highly religious populations [16].

In response to COVID-19 challenges, research institutions have expressed the need for a personalized approach to risk prognosis [20,21,22,23], as well as a resource for engagement with vaccination and related studies. Currently, online surveys are being used to report symptoms and to investigate psychological factors related to the pandemic [22,23,24,25,26]. Several large-scale studies have supported online surveys to assess the risk of COVID-19 and have advised governments about self-isolation, testing, vaccinations, and long-term effects [27,28,29,30]. Still, authorities do not make full use of all the tools that digital technology offers to improve information dissemination and enhance the discussion of COVID-related problems within large populations. The general public struggles with immense amounts of misinformation. A recent analysis noted that 31 million people follow anti-vaccine groups on Facebook, and 17 million people are subscribed to similar content on YouTube [31]. The WHO has warned that despite the best efforts to promote vaccine awareness, misinformation has led to an infodemic about COVID-19 spreading online [32]. Many post-COVID initiatives aim to approach these issues with human-centricity in mind and ensure transparency in acquiring, storing, and using real-world data by taking people’s and societies’ interests as a guiding principle [33].

The use of digital technology is still not sufficient to strengthen pandemic management and preparedness for possible future disease outbreaks [33,34]. Most existing technological solutions that assess the risk of being infected by COVID-19 are designed for individual use only and suffer from an insufficient amount of information for future use in scientific research and for decision making [24,34]. Digital technologies can help scientists and governments to reach large audiences and provide reliable information about COVID-19 and vaccination strategies [23,33].

In this study we attempted to provide a technological solution that would allow us to reach a large number of participants in a short time. For this purpose, we predefined a six-week period to evaluate the number of people that would use the platform during this time. We further sought to investigate the factors that affect the vaccination readiness of participants and their COVID-19-related behavioral patterns. In particular, we explored whether those who have less fear of COVID-19 also have a greater risk of infection and less readiness to vaccinate than those who take the threat more seriously.

The aims of the study were:

1. To provide an efficient technological solution (digital platform) for communication between scientists and the general population to discuss personalized risk factors for severe COVID-19 disease.
2. To use this technological solution to investigate the association between vaccination readiness and the risk of COVID-19 infection or severe disease among the population of Latvia within an exploratory study.

## 2. Materials and Methods

### 2.1. Development of the Digital Platform

In order to promote public participation in scientific initiatives and to collect individual information on study participants, it was proposed to use a digital engagement platform for public involvement in research [34]. The digital engagement platform was developed as a collaboration among clinicians, epidemiologists, data protection specialists, and digital health specialists. It is a web-based and mobile-ready app, engaging participants to take part in COVID-19-related surveys and receive risk assessments and specialists’ recommendations. The website of this study includes detailed instructions in Latvian and Russian languages on how to participate in the study, information regarding the funding and organization of the study, and a contact section. Additionally, it offers a Questions and Answers module covering the most commonly asked questions on the content of the study. Participants were first-time platform users, recruited on a ‘first come, first served’ basis. Communication was done using regional media channels and through social media marketing. Once a participant provided consent and engaged in data collection activities, the system extrapolated a meta-dataset that described the user survey input results, fully separated from the actual database.

### 2.2. Exploratory Study

#### 2.2.1. Study Design and Population

The exploratory study was conducted in Latvia, in both Latvian and Russian, and included participants from all regions of the country. The newly developed digital platform was used for enrollment [34]. Participants, who had to be older than 18 years of age, were asked to complete a survey. The survey’s introduction detailed that participation was anonymous and stated that the respondents’ voluntary completion of the survey indicated their consent. Based on a preliminary check by our staff, completion of the survey required approximately 8 min.

Upon completing the survey, participants received COVID-19 risk assessments and recommendations from field professionals to reduce the risk of infection. We asked specialists from different fields to provide basic recommendations regarding the best behavioral patterns to avoid infection by COVID-19. Medical doctors, nutrition specialists, a physiotherapist, an epidemiologist, and a psychotherapist provided recommendations about nutrition, physical activity, COVID-19 prophylactic measures, and behavior. These recommendations were largely standardized (Appendix A). However, they were personalized to some extent, as the recommendations provided to each participant focused on the most problematic behavior mentioned in the survey.

Participants were able to review their responses and fill out the questionnaire repeatedly (for example, to correct errors or to update information that had changed). The study was approved by the Scientific Research Ethics Commission of the Institute of Cardiology and Regenerative Medicine, University of Latvia (20 January 2021).

The survey had two different modules: one assessed COVID-19-related risk of infection and risk of having a severe disease, and the second assessed vaccination readiness. We analyzed only the answers of those participants who completed both modules.

#### 2.2.2. Statistical Analysis

Questions in the survey covered four areas: demographics (9 items), COVID-19-related behavioral factors that could increase the risk of infection (15 items), personal risk factors that could increase the risk of severe disease (10 items), and reasons for vaccination (10 items) (Appendix B).

Vaccination readiness was assessed as no/maybe/yes and further combined for the logistic regression model into no/yes categories by merging the maybe and no responses. From the perspective of public health authorities, people who are uncertain about getting a vaccination should be treated exactly as those who are not willing to be vaccinated at all [15]. We therefore decided to combine the two groups in our analysis. From all the factors that related to the possibility of being infected by COVID-19 (Body Mass Index (BMI), social distancing, type of transport used, profession, work conditions, use of disinfecting solutions, disinfection of surfaces, having chronic diseases, etc.), we calculated the risk of infection using formula (1) and the risk of severe disease using formulas (2) and (3), by adapting the risk calculation and severity model proposed by Chatterjee et al. [35], as well as by Jin et al. [36]:

Risk of Infection = 0.6 ∗ ((Transport + Work + Home + Home Density + Profession + Public Events + Travel)/6) + 0.4 ∗ ((Social Distancing + Face Mask + Use of Disinfection Solution + Disinfection of Surfaces)/4).(1)

For a female:

SEVERITY = 0.3 ∗ (Age/1) + 0.2 ∗ ((Gender + Pregnancy)/2) + 0.2 ∗ ((BMI + Smoking Status)/2).(2)

For a male or unknown:

SEVERITY = 0.3 ∗ (Age/1) + 0.2 ∗ (Gender/1) + 0.2 ∗ ((BMI + Smoking Status)/2). 
Then, Severity Percentage = ((SEVERITY ∗ 0.95)/4.6) ∗ 100. (3)

The risk of infection was calculated according to Chatterjee et al. [35], adapting the coefficients to the situation in our country. We calculated the risk of infection and the severity independently and adapted the coefficients of Chatterjee et al. accordingly. We calculated the mean value of each parameter (exposure and behavior) that was included in the risk calculation formula. We additionally changed part of the variables not relevant for Latvia (for example, residential type). For the severity, we used the main risk factors mentioned by Jin et al. [36], calculating the mean value for each group of factors and weighting each group of factors the weight according to that study.

Descriptive statistics were determined separately for all study participants. Group variables were described with the number of participants and percentage in each group, and continuous data were described with the median and interquartile range (IQR) according to their distribution.

We performed conventional univariate analyses to evaluate relationships between vaccination readiness and socio-demographic data, risk of infection and severe disease, and factors affecting the participants’ motivation to be vaccinated. The significance level at this stage of analysis was *p* ≤ 0.05. Multiple logistic regression models were built to investigate the most important factors associated with vaccination readiness. The full adjustment set included education, anxiety, risk of infection, risk of severe disease, and factors affecting the motivation to get a vaccine that displayed statistical significance in the univariate stage of analysis. We described results of multiple regression models using odds ratios (OR) and 95% confidence intervals (CI).

For the sensitivity analysis, we calculated the overall motivation of participants to be vaccinated by summarizing ten individual factors affecting their motivation and included them in the logistic regression model. All data were processed using statistical program for social studies (SPSS) software (version 26) [37].

## 3. Results

### 3.1. Structure of the Digital Platform

**Figure 1:**
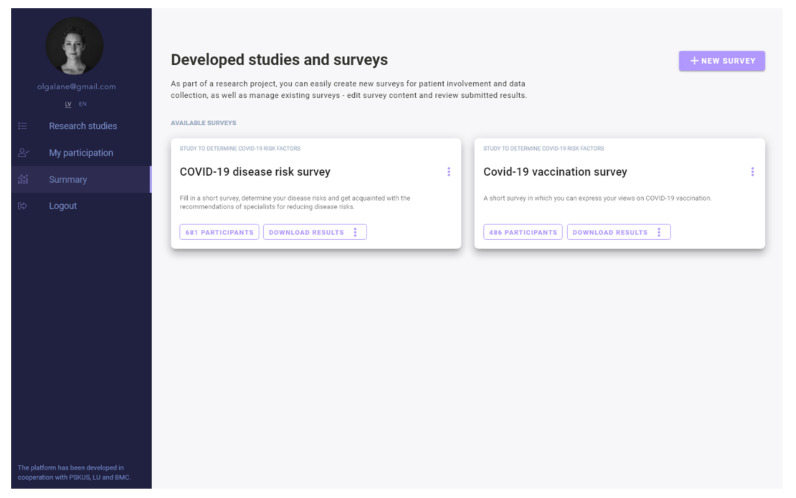
Digital engagement platform.

We developed a digital platform (web- and mobile-ready) [24] that gives researchers the ability to create and manage study projects and to stratify participant cohorts in real time (Figure 1).

**Figure 2:**
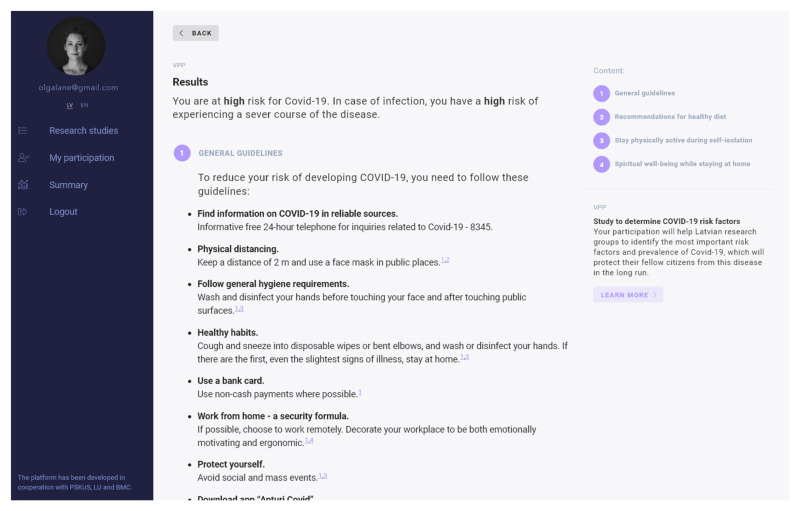
Report received after participation in the survey.

The platform provides a public-facing interface for participants to engage in studies and receive personalized reports (Figure 2).

**Figure 3:**
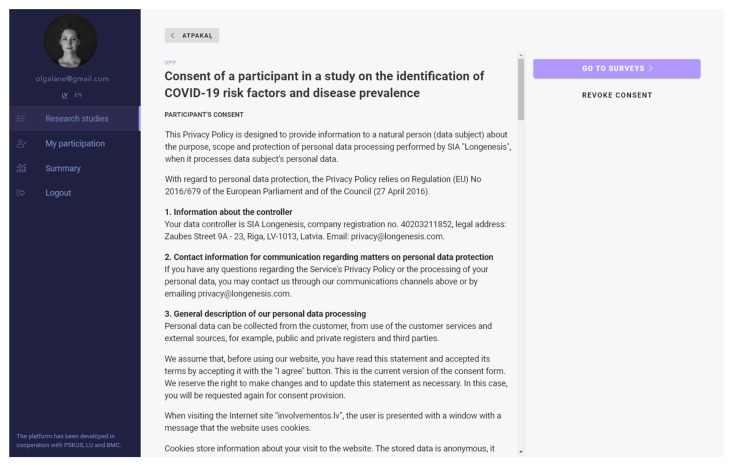
Dynamic consent management.

Dynamic consent management (via the Longenesis Themis API) allows participants to opt out or/and receive invitations for follow-up studies, in compliance with the General Data Protection Regulation (GDPR) (Figure 3) [38].

The population-based cross-sectional exploratory study was performed in Latvia using the described platform.

### 3.2. Results of the Exploratory Study

Data were collected between 1 February 2021 and 10 March 2021. We collected demographic (age, gender), clinical (information on chronic diseases that were previously shown to be related to severe COVID-19 disease), and behavioral information possibly related to the risk of infection.

A total of 677 participants completed the first module of the survey (questions related to the risk of infection or severe disease), and 482 participants took part in the second module, regarding vaccination readiness. In total, 467 participants completed both modules of the survey.

Women made up a significant majority of the participants. Respondents were from 16 to 68 years old; 190 out of 465 (40.7%) were from the age group 25–34 years, mostly from the capital city of Latvia, married or living in a civil union. Most of them did not have children, but five of them were pregnant at the time they took the survey (Table 1). Most of the participants (N = 459 of 465, 98.3%) had not been diagnosed as COVID-19 positive in the past.

Most participants did not have any chronic disease (N = 391 out of 467, 83.7%). Out of all 467 participants, 29 (6.2%) had cardiovascular diseases, 26 (5.6%) had asthma, 18 had immune diseases, 6 (1.3%) participants had liver diseases, 5 (1.1%) participants were oncological, 3 (0.6%) had chronic kidney disease, 2 (0.4%) had diabetes mellitus, and 2 had chronic obstructive pulmonary disease. All participants except one had not traveled in the previous 14 days, most (N = 461 of 467, 98.7%) did not participate in public events, and most (458 out of 467, 98.1%) wore masks whilst going outdoors. Half of the participants used private transport (235 out of 467, 50.3%), and 109 out of 467 (23.3%) worked in health care or had direct contact with a large number of people, but 199 out of 467 (42.6%) mentioned that they worked from home. More than 70% of the participants (346 out of 467) used disinfectants at least sometimes, and 278 out of 467 (59.5%) disinfected surfaces very often. There were 177 out of 467 (37.9%) participants who were not anxious or were sometimes anxious about getting COVID-19. Overall, the calculated risk to get severe disease ranged from 12.4 to 64.0, with a median of 22.7 (IQR 16.5–28.9). Most participants had a medium risk of infection (N = 224 out of 476, 48%) but a low risk of severe disease (N = 324 out of 467, 69.4%).

Most of the 467 participants (N = 401, 85.9%) had a readiness to take the vaccine against COVID-19; 20 participants (4.3%) mentioned that they did not want to be vaccinated, and an additional 46 participants (9.9%) were hesitant. There was a significant relationship between readiness to take the vaccine and giving recommendations to take the vaccine to relatives and friends (*p* = 0.01); however, of those who would refuse to take the vaccine (N = 20), 7 people (35.0%) said they would either recommend or probably recommend it to others.

Risk factors for infection or severe disease that were related to vaccination readiness at the 0.05 significance level were: the respondent’s profession, the use of disinfecting solution, frequent disinfection of surfaces, and social distancing (Table 2). Of the ten factors affecting the motivation to be vaccinated, only two were univariately related to vaccination readiness: former sickness from COVID-19 and a free vaccine (data not shown).

In the fully adjusted multiple logistic regression models, the level of anxiety was the most important factor associated with readiness to take the vaccine. Of all the factors that were related to the motivation to be vaccinated, trust in pharmaceutical companies and feelings of social responsibility were significantly associated with vaccination readiness (Table 3).

Overall motivation to be vaccinated ranged from 10.0 to 50.0, with a median of 37.0 (IQR 32.4–45.3). In the sensitivity analysis, overall motivation increased vaccination readiness (OR = 1.44, CI 1.09; 1.20). Other associations in the model did not change.

## 4. Discussion

In six weeks, we engaged more than 600 participants. This confirms the fact that people are motivated to collaborate, and digital solutions can make it possible to reach a large audience in a short time. In line with our research, other studies previously have shown online surveys to be useful in reaching the general public and in overcoming biases created by national and regional policies [24,39]. However, like other similar online risk assessment tools [36,37], our digital platform is specifically designed to comply with local health policy and interventions to manage disease-related risks. In this exploratory study, we find that the platform is an effective instrument for promoting health and establishing communication between scientists and communities.

The second aim of our study was to use digital outreach to investigate the association between vaccination readiness and the risk of COVID-19 infection or severe disease. We found three factors were associated with vaccination readiness: the participants’ level of anxiety, their trust in pharmaceutical companies, and their feelings of social responsibility. Similar results were observed by Benis [40]. Interestingly, risk of infection and risk of severe disease were not found to be associated with vaccination readiness. Yet the most prominent factor in increasing vaccination readiness was the participants’ level of anxiety. These two results suggest that people do not understand that vaccination will reduce their risk of infection and severe disease, which in turn indicates that public health authorities need to provide fuller explanations.

Studies on vaccine hesitance performed in different countries came to the same list of major factors that affect vaccination readiness. First, a lack of appropriate information was identified as a major factor for 27% of those that hesitate to be vaccinated [15]. Participants that want to get a vaccine were five times more informed on how the vaccines work and their role in stopping the pandemic than those that were not ready to get a vaccinated [14]. Socio-demographic disparities may be another factor that affects vaccination readiness. For example, a recent large-scale study by Hyland et al., (2021) which was performed in the United Kingdom and Ireland found that those that are “vaccine deniers” were typically younger, with a lower income and mostly living alone [41]. Female gender and living in disadvantaged areas were found to be a major feature of the 29% of vaccine hesitant participants in an Australian study by Edwards et al., (2021), with 7% of participants being vaccine resistant [42]. Overall, most of the studies agree that successful, and customized communications with each segment of the population can lead to higher rates of vaccination. In a study performed in the Israeli population [39], authors attempted to identify a possible link between seasonal vaccination against influenza, and social media engagement and perception of reliability during the COVID-19 pandemic. Questions from the questionnaire covered aspects of socio-demographics, social media usage, influenza and vaccine-related knowledge and behavior, health-related information searching and publishing, its reliability, and its influence, as well as COVID-19- and vaccine-related information searching and publishing, its reliability, and its influence. Data analysis on 207 participants showed that women and younger people in general were more actively involved in the study. Vaccinated individuals were more active and involved in their health management, whereas fewer social media users were vaccinated against influenza. It also indicates that health communication countermeasures must be developed and adapted over all communication channels to reach the target public who have low confidence in the information disseminated by governmental and health organizations.

The other study by Benis et al. [40] investigated the reasons for disparate attitudes towards vaccination and identified socio-demographic and other key attributes affecting COVID-19 vaccination adherence in the US population. The questions in the questionnaire were focused on the intent and reasons to take and recommend the COVID-19 vaccine, the person’s health status regarding the COVID-19 disease and socio-demographics. Data from 1644 answer sets shows the participants were mainly women and the median participant’s age roughly matched the US population’s median age. The participants answering that they intended to get the vaccine had a higher education level, more children and were mostly from the white population. The differences in opinions regarding civic responsibility were critical factors in vaccine acceptance (87%) and confidence in the pharmaceutical industry (74%) had a positive impact on vaccination adherence. The fear of COVID-19 was a major factor in intent to be vaccinated.

In our study, trust in pharmaceutical companies increased vaccination readiness, but trust in government and trust in the health care profession did not affect vaccination readiness. Another factor that did not affect vaccination readiness was the level of education, regardless of the fact that most participants in this exploratory study had a higher education. With the rise of public interest and concerns around the new COVID-19 vaccines, the public health and scientific authorities need to understand which factors influence vaccination compliance [43] and which of them do not, so that they can tailor their efforts to reach large communities and persuade people to get the vaccine. Large-scale distribution of a COVID-19 vaccine will require sufficient health system capacity, as well as strategies to enhance trust in and acceptance of the vaccine and those who deliver it [44]. The COVID-19 pandemic is expected to continue to impose enormous burdens of morbidity and mortality while severely disrupting societies and economies worldwide. An approach to calculate the risk of infection and severity of COVID-19 among the population will make it possible to prioritize high-risk populations for vaccination and will help to maximize the number of lives saved [45]. A major part of the response to the pandemic depends on culture and citizen behavior. The results of an ecological study by Gelfand et al. investigating the link between COVID-19-related mortality and cultural tightness in 57 nations concluded that interventions to effectively mitigate COVID-19 must fit countries’ cultural norms to be successful [45]. However, previous experience in changing social norms may determine the winning strategies against the pandemic and other collective threats. For example, it might be reflected in the fast and effective vaccine distribution in Israel (4.65 million doses) and the USA (66 million), although both countries were defined as moderate-to-loose cultures in the paper mentioned above [46]. Nevertheless, in our study, we observed that although the Latvian population tends to be moderate in cultural tightness and to generally comply with social policy measures, people who do not follow the recommendations of public health authorities mostly have a medium to high risk of infection. The authorities should plan proper strategies to fight such a low level of compliance, as studies show that following the recommendations of public health authorities is a major factor in reducing COVID-19 morbidity in populations. For example, in the study by Rader et al., masks were shown to reduce pathogen spread for all levels of physical distancing [27].

Most of our study participants were young. As in other studies on the use of digital technology, we find that older populations are less involved in digital surveys [47,48]. This fact means that authorities should provide separate advertisements for older people to motivate them to participate in the dialogue. In addition, this fact can indirectly point to a need to increase digital knowledge among the elderly, as mentioned in other studies [49]. Similarly to other studies, our digital platform [50] highlighted the fact that women more actively participate in any kind of assessment (about two-thirds of the participants in this exploratory study were women). However, men have been found to have a higher risk of severe COVID-19 disease [36]. These two factors—a disproportion in the age of participants towards younger ones and a higher percentage of women in the study population—can lead to underestimation of the global risk of infection in the entire population and should be considered whilst planning public health strategies.

The study that we performed for the “hottest” theme nowadays, was in our case, an exploratory one for the use of a digital engagement platform in our country, and we proved its effectiveness in these circumstances. However, we assume that in different countries and discussing different study questions the effectiveness of such an instrument could differ. We suggest performing a short exploratory study for each population when considering the use of similar platforms.

### Limitations and Strengths of the Study

The epidemiological, exploratory part of our study has some limitations. First, there is likely under-sampling of several groups that were unable to take part or uninterested in participating in online surveys, including groups that lack digital skills. Another limitation of this study is its cross-sectional nature, which does not allow us to make proper conclusions on risk factors for COVID-19 infection or severe disease in this specific population, thereby probably underestimating the association of these risks with vaccination readiness.

The short duration of the study and the relatively small number of participants are also limitations. We observed higher interest in the module related to the risk of infection than in the module related to vaccination, and this is another limitation of our study. Moreover, as all answers were self-assessed, information bias is a possible limitation of the study. Finally, this exploratory study was performed in one specific population in Latvia, and this population might differ from other groups. However, the major strength of this study is that it provides an example of using digital technology to establish contact between scientists, authorities, and the broader population that can serve as a model for deploying such technology for scientific needs around the world.

## 5. Conclusions

While the pandemic is global, the public response is local and specific, so sufficient effort needs to be invested to enable a change in the public perception of the disease and to create more compliance with risk-prevention behavior. Digital platforms are appropriate instruments to open a dialogue on COVID-19-related issues and vaccination in large populations. We have recommended that the Ministry of Healthcare and the Ministry of Science and Education of the Republic of Latvia use a digital platform to communicate the results of scientific projects to the public. To that end, they should develop guidelines for public participation in research projects on pandemic topics, including data reuse in research, and embrace dynamic human consent management to control the use of personal data.

The use of digital technologies for connecting state authorities, physicians and appropriate segments of the population can be an effective solution in times of pandemic and after the mitigation of the current crisis. The digital polling of a population’s opinion and key motives for noncompliance with medical recommendations can be important in multiple cases of hesitancy for primary prevention strategies, such as the avoidance of mammographic testing, the low public interest in colonoscopy screening and others. Digital polling technologies could be widely implemented around the world to overcome public barriers that hinder the effectiveness of various public health initiatives.

## Figures and Tables

**Table 1:** Socio-demographic data of study participants who completed both surveys.

Variable	Category	N = 467
Gender, N (%)		333 (71.3)
		5 (1.1)
Age, median (IQR)		34.0 (28.0–42.0)
Marital status, N (%)	Single	121 (25.9)
	Married/civil union	306 (65.5)
	Separated or divorced	17 (3.6)
	Widowed	4 (0.9)
Number of children, N (%)	0	238 (51.1)
	1	88 (18.8)
	2	86 (18.5)
	3	38 (8.2)
	4 and more	11 (2.4)
Education, N (%)	Less than high school	5 (1.1)
	High school	116 (24.9)
	Bachelor’s degree	147 (31.5)
	Master’s degree	193 (41.4)
Location	Capital city	289 (62.3)
	Other cities	49 (10.6)
	Rural areas	126 (27.2)
Type of dwelling	Apartment	358 (76.7)
	Private home	104 (22.3)
	Dormitory	4 (0.9)
	Nursing home	1 (0.2)
Number of people at home, median (IQR)		2.0 (2.0–4.0)
Number of rooms, median (IQR)		3.0 (2.0–4.0)

**Table 2:** Risk factors of infection or severe disease by vaccination readiness.

		Vaccination Readiness		
Variable	Category/Factor Affecting Motivation	Yes N = 401 (85.9%)	No/Not Sure N = 66 (14.1%)	*p*
Smoking status, N (%)	Never	203 (84.9)	36 (15.1)	0.59
	Not a smoker < 2 years	42 (82.4)	9 (16.4)	
	Smoker < 5 years	26 (92.9)	2 (7.1)	
	Smoker ≥ 5 years	56 (87.5)	8 (12.5)	
BMI (kg/m^2^), median (IQR)		23.9 (21.5–27.6)	24.8 (22.1–29.7)	0.24
Having chronic disease, N (%)	No	334 (85.4)	57 (14.6)	0.59
	Yes	67 (88.2)	9 (11.8)	
Transport, N (%)	Stay at home	55 (88.7)	7 (11.3)	0.09
	Walking	96 (88.9)	12 (11.1)	
	Private transport	203 (86.4)	32 (13.6)	
	Public transport	47 (75.8)	15 (24.2)	
Profession, N (%)	Health care	51 (98.1)	1 (1.9)	0.05
	Direct with people	46 (80.7)	11 (19.3)	
	Indirect with people	23 (85.2)	4 (14.8)	
	Other	281 (84.9)	50 (15.1)	
Use of disinfecting solution, N (%)	No	92 (76.0)	29 (24.0)	<0.01
	Sometimes	158 (89.3)	19 (10.7)	
	Yes	151 (89.3)	18 (10.7)	
Disinfection of surfaces, N (%)	Very often	250 (89.9)	28 (10.1)	<0.01
	Often	132 (86.8)	20 (13.2)	
	Sometimes	19 (51.4)	18 (48.6)	
Social distancing, N (%)	No/sometimes	53 (75.7)	17 (24.3)	0.01
	Yes	348 (87.7)	49 (12.3)	
Anxiety, N (%)	Never	9 (40.9)	13 (59.1)	<0.01
	Sometimes	118 (76.1)	37 (23.9)	
	Often	171 (92.4)	14 (7.6)	
	Very often	103 (98.1)	2 (1.9)	
Risk of infection, median (IQR)		36.7 (32.4–45.3)	38.9 (34.5–45.3)	0.08
Risk to get severe disease, median (IQR)		22.7 (16.5–28.9)	24.8 (18.6–31.0)	0.05

**Table 3:** Association between vaccination readiness and socio-demographic factors, motivation, and risk.

Variable	Factors	Odds Ratio, OR	95% Confidence Interval, CI	*p*
Education		1.26	0.82; 1.93	0.29
Anxiety		3.09	1.88; 5.09	<0.01
Risk of infection		1.01	0.97; 1.06	0.63
Risk of severe disease		0.99	0.96; 1.03	0.66
Factors affecting the motivation to be vaccinated	Fear of COVID-19	1.23	0.91; 1.68	0.18
	To protect family	0.81	0.54; 1.20	0.29
	Trust in health care	1.18	0.80; 1.75	0.41
	Trust in pharmaceutical companies	1.53	1.03; 2.27	0.03
	Innovative vaccine	1.02	0.72; 1.45	0.89
	Employer recommendation	1.22	0.92; 1.62	0.16
	Trust in government	1.14	0.81; 1.62	0.45
	Social responsibility	1.61	1.16; 2.22	<0.01

## Data Availability

Public health practitioners and research institutions can request access to metadata via the Longenesis Curator platform, according to FAIR data principles [39].

## Appendix A. Standardized Recommendations for Study Participants

You are at high risk for COVID-19. In case of infection, you have a high chance of experiencing a severe course of the disease.

1. GENERAL GUIDELINE

To reduce your risk of developing COVID-19, you need to follow these guidelines:

- Seek information on COVID-19 in reliable sources. Informative free 24-h telephone for inquiries related to COVID-19: 8345.
- Physical distancing. Keep a distance of 2 m and use a face mask in public places.
- Follow general hygiene requirements. Wash and disinfect hands before touching the face and after touching public surfaces.
- Healthy habits. Cough and sneeze into disposable wipes or bent elbow, and wash or disinfect your hands. If there are the first, even the slightest, signs of illness, stay at home.
- Use a bank card. Use non-cash payments as much as possible.
- Working from home is a security formula. If possible, choose to work remotely. Arrange your workplace to be both emotionally motivating and ergonomic.
- Protect yourself. Avoid social and mass events.
- Download the Stop COVID app. The app is the fastest way to find out if you have been in contact with a COVID-19 carrier. Do not remain ignorant, and make informed decisions. Find out more at https://www.apturicovid.lv/#en (accessed on 18 November 2021).

If you are over 60 years of age and/or have a chronic illness, please follow these guidelines in addition to the guidelines above:

- Avoid sick people.
- Shop when the store is not crowded or ask someone to do it for you.
- Avoid public transport during traffic congestion.
- Exercise outdoors.
- Get medical attention immediately if you notice any symptoms of COVID-19.

2. RECOMMENDATIONS FOR HEALTHY NUTRITION

Proper nutrition is crucial for health, especially when the immune system may have to fight viral illnesses. Limited access to fresh food can jeopardize the ability to continue eating a healthy and varied diet. It can also potentially lead to the consumption of highly processed foods, which are usually high in fat, sugar, and salt. Nevertheless, even with a few and limited ingredients, a person can create a diet that maintains strong health.

- Cook at home, plan your purchases in time—create your menu!
- The right strategy for creating a menu—prioritize fresh produce.
- Be aware of the portion size of the food!
- Control your salt and sugar intake, and as much as possible avoid trans fats.

3. STAY PHYSICALLY ACTIVE DURING SELF-ISOLATION

Physical activity and relaxation techniques can be valuable tools to help you stay calm and continue to protect your health during this time. The WHO recommends 150 min of moderate-intensity or 75 min of intense physical activity per week, or a combination of both. These recommendations can still be achieved even at home, without special equipment, and with limited space. Here are some tips for staying active and reducing sedentary behavior while at home in quarantine:

- Take short active breaks during the day. Dancing, playing with children, and doing housework like cleaning and gardening are just some of the ways to stay active at home.
- Attend sports classes online. Take advantage of the available online gym classes. Many of them are free and can be found on YouTube. Before trying them, carefully evaluate your abilities!
- Take a walk. Even in small spaces, walking around or walking on the spot can help keep you active.
- Get up. Reduce sedentary time whenever you get up. Ideally, try to interrupt your sitting time every 30 min.

4. MENTAL WELL-BEING AT HOME

Taking care of your mind as well as your body is really important if you stay at home because of the coronavirus (COVID-19). You may feel depressed or worried about your finances, your health, or your loved ones. Maybe you feel bored, frustrated, or lonely. It is important to remember that it is normal to feel this way and that each person reacts differently. Remember, most of us will experience these feelings. Staying at home can be difficult, but by doing so, you help protect yourself and others. There are things you can do now to help maintain mental well-being and deal with how you might feel when you are at home. Make sure you get extra support if you feel you need it.

- Pause. Inhale. Reflect. Inhale slowly through your nose, then exhale slowly. Such breathing is one of the best ways to relieve stress, as it signals to your brain the need for rest.
- Connect with others. Contact people close to you regularly. Tell them how you feel and share your thoughts.
- Limit the time you spend reading the news. Better use this time to relax from your computer screen or take a walk in the fresh air.
- Schedule time for hobbies. If we feel anxious, lonely or low, we can stop doing things that we normally enjoy.

5. REFERENCES

[1] https://likumi.lv/ta/id/314641-ieteikumi-covid-19-infekcijas-profilaksei (accessed on 18 November 2021).
[2] https://www.spkc.gov.lv/lv/masku-lietosana (accessed on 18 November 2021).
[3] https://www.ecdc.europa.eu/en/covid-19/prevention-and-control/protect-yourself (accessed on 18 November 2021).
[4] https://www.spkc.gov.lv/lv/darba-devejiem (accessed on 18 November 2021).

## Appendix B

**Table A1:** List of Questions Presented to the Study Participants.

Area of Investigation	Questions	Explanation of Measures
Demographic and anthropometric questions (9 items)	What is your age group (in years)? What is your gender? What is your marital status? How many children do you have? What is your education level? What is your district of residence? Are you currently pregnant? What is your weight? What is your height?	7 groups with 10 years range 4 categories 5 categories Range 0–4 and more 4 categories (from less than high school to postgraduate) List of Latvia districts Pregnancy status (yes/no) Kg Cm
COVID-19-related behavioral factors that could increase the risk of infection (15 items)	What is your smoking status? Duration of cigarette smoking When did you quit smoking? Which way of transport do you use? What are your working conditions? Do you comply with social distancing? Do you use face mask? How often do you disinfect your hands? Do you disinfect your hands before touching your face? How much do you worry about COVID-19? What are your living conditions? What is your profession? What are your thoughts about the existing COVID-19 restrictions? Did you in last 14 days travel abroad or visit mass events? What is in your opinion of the percentage of people that follow the official state COVID-19 restrictions?	4 categories Years Years 4 items 7 items Yes/no 3 categories 3 categories 3 categories 3 categories 4 categories of living density 4 categories, increased risk of infection in workplace 3 categories, attitude 4 answers 3 categories (from < 50% to > 80%)
Personal risk factors that could increase the risk of severe disease (10 items)	Do you have chronic diseases?	10 items (yes/no)
Reasons for vaccination (10 items)	Please explain your motivation to vaccinate or not: • Fear of COVID-19 • To protect my family and our relatives • Confidence in our healthcare providers • Confidence in our pharmaceutical industry • The COVID-19 vaccines are revolutionary and use innovative technology • Employer recommends/demands • Confidence in governmental leadership’s guidance • It is my civic responsibility to take this vaccine • Myself or relatives got sick with COVID-19 • Free of charge	Scaled from 1 (completely disagree) to 5 (completely agree)
